# PHY·FI: fast and easy online creation and manipulation of phylogeny color figures

**DOI:** 10.1186/1471-2105-7-315

**Published:** 2006-06-22

**Authors:** Jakob Fredslund

**Affiliations:** 1 Bioinformatics Research Center, Aarhus University, Høegh-Guldbergs Gade 10, Bldg. 1090, DK-8000 Århus C, Denmark

## Abstract

**Background:**

The need to depict a phylogeny, or some other kind of abstract tree, is very frequently experienced by researchers from a broad range of biological and computational disciplines. Thousands of papers and talks include phylogeny figures, and often during everyday work, one would like to quickly get a graphical display of, e.g., the phylogenetic relationship between a set of sequences as calculated by an alignment program such as ClustalW or the phylogenetic package Phylip. A wealth of software tools capable of tree drawing exists; most are comprehensive packages that also perform various types of analysis, and hence they are available only for download and installing. Some online tools exist, too.

**Results:**

This paper presents an online tool, PHY·FI, which encompasses all the qualities of existing online programs and adds functionality to hopefully eliminate the need for post-processing the phylogeny figure in some other general-purpose graphics program. PHY·FI is versatile, easy-to-use and fast, and supports comprehensive graphical control, several download image formats, and the possibility of dynamically collapsing groups of nodes into named subtrees (e.g. "Primates"). The user can create a color figure from any phylogeny, or other kind of tree, represented in the widely used parenthesized Newick format.

**Conclusion:**

PHY·FI is fast and easy to use, yet still offers full color control, tree manipulation, and several image formats. It does not require any downloading and installing, and thus any internet user regardless of computer skills, and computer platform, can benefit from it. PHY·FI is free for all and is available from this web address:

## Background

Many researchers from a wide range of biological and computational disciplines have a recurrent need for depicting trees of various forms, e.g. phylogenies. Often, the tree is represented as a text string or file in the parenthesized Newick format. This is true for phylogenies produced by software such as the Phylip package [1] or ClustalW [2]. For example, when performing a multiple alignment of sequences with ClustalW, apart from the alignment itself a dendrogram file is produced with extension .dnd, and this file contains a Newick representation of the phylogeny inferred from aligning the sequences.

From the rather incomprehensible textual description of a Newick tree, humans can learn little about the overall tree structure or of the relationship between particular nodes:

(Bovine:0.69395,(Gibbon:0.36079, (Orangutan:0.33636,(Gorilla:0.17147, (Chimpanzee:0.19268,Human:0.11927):0.08386):0.06124):0.15057):0.54939,Mouse:1.2146);

Hence, for full comprehension of the tree structure, researchers will need a graphical image of the tree to benefit from the information it encodes. This paper presents PHY·FI, a new tool for drawing such Newick trees. The strengths of PHY·FI are these:

• It is web based; i.e., no need to download and install anything. It works from any computer connected to the internet, and the user has the tree in seconds, displayed in the browser.

• The tree can be manipulated directly in the browser: Nodes (subtrees) can be collapsed and expanded.

• The image can be downloaded in the most common formats. (By request, new formats can easily be added).

• The user-interface is self-explanatory.

• The user has full color control over nodes, labels, etc.

• Several other graphical parameters can be modified by the user.

• The user can redraw the image and immediately observe the changes after tuning the parameters.

There is a wealth of existing software programs which can draw phylogenies. Some of these programs are full phylogenetic inference packages which include the facility to draw trees, while others are developed specifically for tree drawing. Before going into depth with PHY·FI, I will give a brief overview.

Most existing programs are for download and local installation only. Some are very elaborate and allow user post-processing through interaction and reshaping of the tree via mouse clicks in the produced image; others are more straight-forward and have fewer degrees of freedom. Sophisticated programs for download and local installation include TREEGRAPH [3], Bosque [4], ATV [5], TREEVIEW [6], NJplot [7], MEGA [8], Mesquite [9], Phylodraw [10], TreeIllustrator [11] and the commercial programs Paup [12], MacClade [13] and TreeMe [14]. Arbor 3D [15] and Walrus [16] are tools for visualizing and interacting with trees in three dimensions. A simpler program, also for download, is DrawTree [17] by T. Mailund.

The elaborate program TreeWiz [18] can handle huge trees with up to 50,000 leaves and lets the user explore such trees by collapsing and expanding nodes interactively. There is an online version, too (which has not been maintained and currently seems not to be functional, but the author offers to update it upon demand): In order to explore one's own trees rather than the given example trees, one must give read access to one's home directory to the program Java applet (in general, this would constitute a somewhat liberal security policy) and create a new directory with a specific name which must hold the trees.

It is also possible to create phylogeny figures from within several programming environments such as the statistical computing programming language R [19], the text processing system LaTeX through G. Savva's Newick tree package [20], and BioPerl using the cladogram drawing module developed by G. Valiente [21].

For all of the above, one needs to at least do some downloading and installing – and in many cases one needs to write computer code – to draw a phylogeny. A few programs, however, do have a web interface and are as such directly comparable to PHY·FI. Phylodendron by D. G. Gilbert [22] presents the user with several choices of tree style and appearance as well as different formats for the output image. Not all combinations of parameter settings seem to be fully implemented, though. The images are in black and white and have no branch length labels (only a rule).

V. Veeramachaneni has written a program with the name TreeDraw [23]. It presents a simple user interface, although one needs to manually type in a color encoding if one wants the leaves in the tree to be colored. The program can produce images in the PS, PDF and PNG formats, but again branch lengths have to be judged by visual inspection with the help of a ruler. The user has no control over the image size and cannot paste a Newick string directly on the web page.

Drawtree [24], written by R. Ree, also has a web interface where you can paste a Newick string and create a black and white PDF or EPS image with little control over the result.

J. Felsenstein's Phylip software [1] also includes the web tool Drawtree [25]. This program lets the user draw black and white images of trees in the PS format and numerous other printer-friendly formats; however, branch length labels seemingly cannot be added to the trees, and the resulting tree image is not immediately shown but has to be downloaded and viewed with some other appropriate application.

T-Rex [26] is an elaborate analysis program which offers sophisticated functionality in terms of, e.g., tree inference. When it comes to displaying trees, it offers several different graphical tree shapes, it allows limited control over the coloring of labels and branches (colors are chosen from a fixed list), and per default it cleverly scales the image to fit the browser window. However, the user cannot upload a Newick tree file but has to paste the string in a text field directly (which is less than optimal for large trees); there is no control over font or font size; branches seem to be drawn with the same length even though in the Newick string, all nodes must have given (not necessarily identical) lengths in order for the parser to work; and finally, not all correctly formatted Newick strings seem to pass the parser.

Lastly, PhyloView [27] is a web based visualization tool which can illustrate the potential differences between the deduced phylogeny of a protein sequence alignment and the taxonomic relationship of the species of the sequences. The user uploads a Newick tree and can then decide any taxonomic partition for the species and choose a coloring. Thus, the aim of the program is not merely displaying Newick trees, and therefore no further control over the tree image is possible.

### Implementation

**Figure 1:**
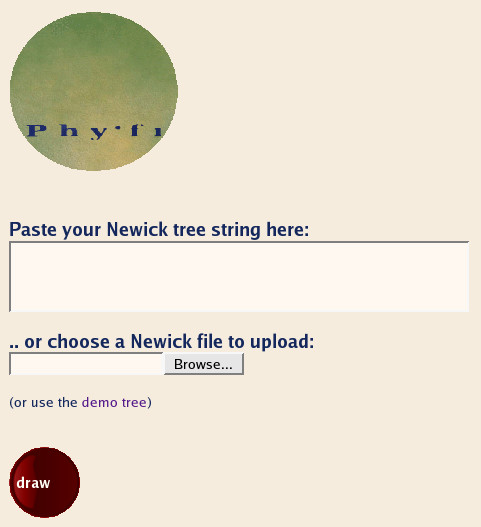
The very straight-forward front page of PHY·FI.

**Figure 2:**
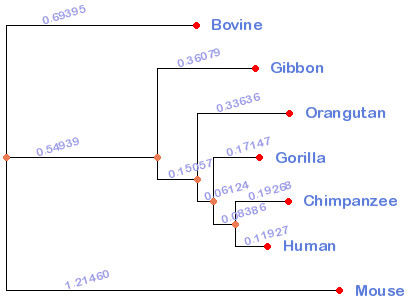
A phylogeny drawn by PHY·FI.

The web based program PHY·FI presented here is an attempt to combine the tree drawing facilities of all these tools into one fast, easy-to-use web tool. On the very simple front page (shown in Figure 1), the user either uploads a Newick tree file or pastes a Newick string directly (or chooses the available demo file), and after clicking the "draw" button, within a few seconds the user is presented with the resulting image. Figure 2 shows the Newick string quoted in the previous section as it may be illustrated by PHY·FI.

**Figure 3:**
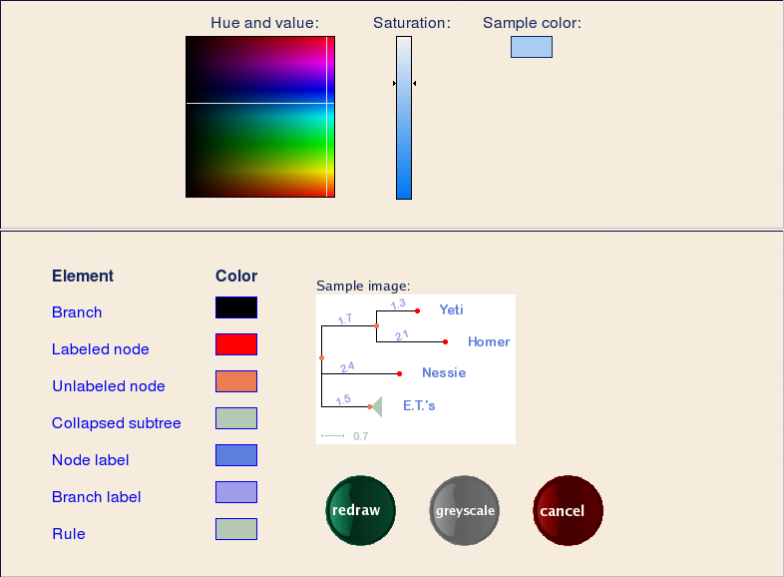
The screen for choosing colors. The user chooses a sample color by clicking in the color palette and saturation bar, then confers this color to some element, e.g. 'labeled node', by clicking its name in the table. The sample image is updated accordingly. To use the new colors, the user clicks the 'redraw' button. Otherwise, (s)he may click the 'cancel' button – or the 'greyscale' button to use greyscale colors.

The tree is drawn left-to-right with leaf nodes on the right, and such that no branches will cross. The image height is initially calculated from the number of leaves to ensure that the labels will be readable – for large trees, this leads to large images, and of course the user may choose to change the image height (as described below). The length of a branch is written directly beside it at an angle which can be set by the user; the user may also choose to instead indicate branch lengths by a rule (drawn in the lower left corner) if the branch labels disturb the overall image. The branches, leaf nodes, internal nodes, node labels, branch labels, rule, and collapsed subtrees (described in the next paragraph) all have their own colors which are set to pleasant, readable defaults, but which may be altered by the user. Colors are chosen in a very intuitive way by clicking with the mouse in two color palettes indicating color hue/value and saturation. By the click of one button, the user may also use greyscale colors (as prescribed by some journals). Figure 3 shows a screen shot of the color choice menu.

**Figure 4:**
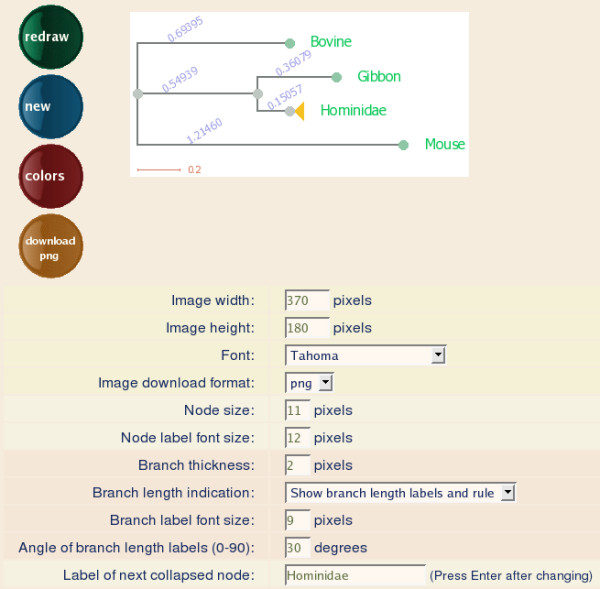
The screen displaying the image and the parameters. Here, the example tree shown in Figure 2 has been re-colored, a rule has been added, and the font and image size have been modified. Further, the root node of the subtree containing the leaf nodes Orangutan, Gorilla, Chimpanzee, and Human has been collapsed and named "Hominidae"; note the yellow triangle marking this collapsed subtree.

After the image is displayed, the user has the opportunity, on the same page, to modify several parameters (as shown in Figure 4). The first group of parameters concerns the image in general: Image width and height (which resizes the tree since the drawing will scale to fit the chosen image size), the font, and the download format (the format of the image file which can be subsequently downloaded to the user's computer; PNG, JPG, GIF, TIF, PDF, EPS). The second group of parameters concerns the nodes: The node size and the node label font size. The third group concerns the branches: Branch thickness, whether and how branch lengths should be shown (by labels and/or by a rule) and the angle in which branch labels are written, and the font size of branch labels. Lastly, the user may choose a name which will be given to the next collapsed node; to collapse and rename a node rooting some subtree, the user types the name to be given to the node in the text field, presses the 'Enter' key, and then clicks on the node directly in the image. This has been done in Figure 4. Collapsed subtrees may be re-created (expanded) by clicking their root nodes again.

Thus, if the image is initially not quite satisfactory, e.g. if some of the node labels appear too close to one another, or if some part of the tree is irrelevant for the user's specific purpose, or if the colors are not quite right, the user simply performs a few modifications by tuning parameters and collapsing nodes, and then clicks the "redraw" button. [See Additional file 1 for more technical details]. After drawing the tree with the desired download format selected, the user clicks the 'download' button to download the image to his/her own computer.

## Results and discussion

PHY·FI has been extensively tested on Windows, Linux, and Mac platforms using Firefox 1.0, Internet Explorer 6.0, Netscape 7.1, Mozilla 1.7.12, and Safari 2.0.3. Trees with up to 3600 nodes were uploaded (for a tree of this size, the image is produced within about 13 seconds). Very large trees yield very large images initially, but the assumption is that the user in such cases intends to collapse parts of the tree in order to get an apprehensible figure out of it. To be able to pinpoint and click specific nodes to collapse them, the user needs to be able to read the labels, and so rather than use some smaller default image height, I have chosen to let the initial height be proportional to the number of leaves in the tree (within a limit of 20,000 pixels, corresponding to an image file size of about 1 Megabyte). The user can always decrease (or increase) the image height and width if (s)he wishes, for instance after creating more empty space by collapsing the irrelevant parts of the tree. Thus, a satisfactory result should always be reachable. Hopefully, the collapse/expand feature may, in conjunction with the regular graphical parameters, relieve the user of any need for post-processing of the image in some other general graphics application.

From the PHY·FI homepage, there is a direct hyperlink to a page describing the Newick standard [28] which the parser expects the input string to follow. According to this standard, internal nodes can be named. Instead of providing string names for (internal) nodes, the user may of course put bootstrap values. PHY·FI does not distinguish between node names and bootstrap values; both are written next to the relevant node in the same color. Thus, to avoid confusion, users should probably not upload input strings in which some nodes have bootstrap values and others have labels.

Several programs, e.g. [22,26], offer various tree drawing styles, such as cladograms and radial trees, rather than just one. PHY·FI only displays trees in one style (phylograms). In the next version, I plan to include several others depending on users' wishes.

## Conclusion

PHY·FI is purely a visualization tool. It has no tree inference capabilities; the user must obtain the Newick string from somewhere else. With this tool I aim to provide other researchers with the possibility of obtaining a readable, aesthetically pleasing and customized tree image in the graphics format of their own choice, all in the matter of seconds or minutes. No download instructions or technical documentation pages stand between the user and the desired phylogeny figure; no extra plug-ins need be installed. The program is meant to be self-explanatory, yet still it presents enough control and interaction to meet most needs. Thus, PHY·FI is very well suited for quickly making a tree figure for, e.g., a paper, a presentation or teaching.

## Availability and requirements

**Project name: **PHY·FI

**Project home page: **

**Operating systems**: Platform independent.

**Programming language: **Python

**License**: PHY·FI is free to all users. Please cite this paper when using images produced by PHY·FI in other publications.

## Abbreviations

Image formats mentioned in the text:

PS: PostScript

EPS: Encapsulated PostScript

PDF: Portable Document Format

PNG: Portable Network Graphics

TIF: Tagged Image File

GIF: Graphic Interchange Format

## Supplementary Material

Additional file 1 About PHY·FI. A short description of PHY·FI plus a few technical details not in the paper on how the trees are drawn. Click here for file
