# Supplementary material for: PHY·FI: fast and easy online creation and manipulation of phylogeny color figures
Source: BMC Bioinformatics. 2006 Jun 22;7:315. doi: 10.1186/1471-2105-7-315 (PMC1513607; doi:10.1186/1471-2105-7-315)
Supplement: Additional file 1 — About PHY·FI. A short description of PHY·FI plus a few technical details not in the paper on how the trees are drawn. [file 1471-2105-7-315-S1.html]

xml version = "1.0" encoding = "UTF-8"?


Phyfi - Online tool for drawing phylogeny figures in color


|  |  |
| --- | --- |
|  | Phy·fi is an online tool which can draw phylogenies and other trees in the Newick format. The philosophy behind it is that it should ..  - Be fast and easy to   use - Supply numerous download formats - Allow the user to collapse/expand nodes - Offer comprehensive color control You supply an input Newick string and draw the tree. Then, if the image looks less than perfect, you can choose another image size to scale the image, toggle the branch length labels on/off or modify the angle with which they're written, choose new colors, collapse an irrelevant subtree by clicking its root, and click redraw to see the effect immediately. Once you're happy with the result, you can download the image in the format of your choice. I hope you find it useful. Please send me an email if you have any comments or suggestions, e.g. if you would like another format to be included in the download options list. Below are some details about how the figure is drawn. Jakob Fredslund  The program parses the input string and creates a tree data structure. Each leaf, and each internal node with a given label, is assigned a unique height (y-coordinate) in the image. This means that as long as the image is made high enough (through the *height* parameter), no labels will overlap. The figure is scaled to "fill" the width and height set by the user. By default, the height is calculated from the number of leaves. In the Newick format, branches may or may not be labeled with a length. In the figure produced by Phy·fi, branches are drawn such that their lengths are as determined in the input string, but the figure is scaled to match the *width* setting. If some but not all branches are labeled in the input string, unlabeled branches are given a default length and drawn as dashed lines in the figure to discrimate them from labeled branches whose length can be "visually trusted". The default length is calculated as the average over the lengths of all labeled branches. If no branches have labels in the input string, all branches in the figure are assigned the same length as appropriate. All branch labels are reformatted such that they have the same number of digits after the comma (in case they have a comma) for easy comparison. Each is written over its corresponding branch starting at a point which is 1/6 of its length from its left end. If the user chooses so, a rule is included in the figure. The rule can be combined with the branch labels, and it can be used as a replacement to increase readability. The length of the rule is found through an intricate algorithm such that it ends up having a length which is approximately 1/7 of the width of the tree, and such that its denomination is a "relevant" number with one significant figure. Go back to the main page |
